# Next-Generation 3D Scaffolds for Nano-Based Chemotherapeutics Delivery and Cancer Treatment

**DOI:** 10.3390/pharmaceutics14122712

**Published:** 2022-12-03

**Authors:** S. M. Shatil Shahriar, Syed Muntazir Andrabi, Farhana Islam, Jeong Man An, Samantha J. Schindler, Mitchell P. Matis, Dong Yun Lee, Yong-kyu Lee

**Affiliations:** 1 Eppley Institute for Research in Cancer and Allied Diseases, College of Medicine, University of Nebraska Medical Center, Omaha, NE 68198, USA; 2 Department of Surgery—Transplant and Mary & Dick Holland Regenerative Medicine Program, College of Medicine, University of Nebraska Medical Center, Omaha, NE 68198, USA; 3 Department of Biochemistry and Molecular Biology, College of Medicine, University of Nebraska Medical Center, Omaha, NE 68198, USA; 4 Department of Bioengineering, College of Engineering, Hanyang University, Seoul 04763, Republic of Korea; 5 College of Medicine, University of Nebraska Medical Center, Omaha, NE 68198, USA; 6 Kansas City Internal Medicine Residency Program, HCA Healthcare, Overland Park, KS 66215, USA; 7 Department of Bioengineering, College of Engineering, and BK21 PLUS Future Biopharmaceutical Human Resources Training and Research Team, Hanyang University, Seoul 04763, Republic of Korea; 8 Institute of Nano Science and Technology (INST), Hanyang University, Seoul 04763, Republic of Korea; 9 4D Biomaterials Center, Korea National University of Transportation, Jeungpyeong 27909, Republic of Korea; 10 Department of Chemical and Biological Engineering, Korea National University of Transportation, Chungju 27469, Republic of Korea

**Keywords:** cancer treatment, chemotherapeutics, 3D scaffolds, materials, polymers, drug delivery

## Abstract

Cancer is the leading cause of death after cardiovascular disease. Despite significant advances in cancer research over the past few decades, it is almost impossible to cure end-stage cancer patients and bring them to remission. Adverse effects of chemotherapy are mainly caused by the accumulation of chemotherapeutic agents in normal tissues, and drug resistance hinders the potential therapeutic effects and curing of this disease. New drug formulations need to be developed to overcome these problems and increase the therapeutic index of chemotherapeutics. As a chemotherapeutic delivery platform, three-dimensional (3D) scaffolds are an up-and-coming option because they can respond to biological factors, modify their properties accordingly, and promote site-specific chemotherapeutic deliveries in a sustainable and controlled release manner. This review paper focuses on the features and applications of the variety of 3D scaffold-based nano-delivery systems that could be used to improve local cancer therapy by selectively delivering chemotherapeutics to the target sites in future.

## 1. Introduction

Cancer is the uncontrolled growth of abnormal cells [1]. The uncontrolled growth may occur in any organ of the body, and its severity may vary from organ to organ (vital or non-vital), as well as among types of cancer and stages of cancer. Treatment of cancers often includes surgery, chemotherapy, radiation, or any combination of them [2]. Despite many advances in cancer treatment over the past few decades, it is still the second leading cause of death worldwide. According to the World Health Organization, the number of deaths from this deadly disease in 2018 exceeded 9.6 million [3]. One of the standard types of therapy for cancer is chemotherapy [4]. Because of the poor bioavailability of chemotherapeutics to cancer cells, chemotherapy requires high doses of drugs that weaken patients’ immunity and cause serious side effects, multiple drug resistance, and poor therapeutic efficacy, leading to higher cancer mortality rates [4,5,6]. As chemotherapeutics travel throughout the body, they kill cancer cells as well as the normal cells of major organs such as the kidneys, lungs, heart, nervous system, and bladder [7]. Therefore, there is a need to develop a chemotherapeutics delivery system that can improve the bio-accessibility of chemotherapeutics in cancer cells through active or passive cellular internalization [2,8].

For therapeutic effectiveness, tumor targeting of the administered drug is necessary, and the desired effect is further enhanced by cell targeting after drug delivery at the tumor site [9,10]. The in situ administration of a drug-loaded scaffold in several types of tumors has been shown to promote the sustainable release of chemo/immunotherapeutics at preselected tumor sites and reduce systemic exposure, in addition to minimizing the frequent repetition of chemotherapy cycles and the economic burden on patients [11,12]. Taking into consideration various forms and stages of cancer, desired drug delivery systems based on an injectable hydrogel/scaffold as a smart approach were produced. These smart delivery systems are majorly stimuli-responsive, such as photosensitive, thermoresponsive, pH-sensitive, and multi-stimuli-based scaffolds [11,13].

Over the years, the knowledge of cancer biology and the increased availability of versatile biomaterials have led to the significant development of nanotechnology and next-generation scaffolds that can deliver drugs to tumor tissue with improved treatment results [14,15,16,17,18,19]. Nanomedicines among the first generation include Doxil^®^ (liposomal doxorubicin) and Abraxane^®^ (paclitaxel bound to albumin), which proved beneficial to cancer patients [20]. Recently, nanoparticles (such as polymeric nanoparticles and liposomes) along with scaffolds presented as 3D networks have gained much attention by enhancing the therapeutic efficacy of drugs both locally and in a targeted manner [21,22,23]. In diseases like cancer, rapid angiogenesis causes fenestrated and leaky vessels, leading to the EPR (enhanced permeability and retention) effect [24]. The EPR effect plays a substantial role in tumor drug delivery, specifically nanoparticles through drug accumulation in tumor sites [24,25,26]. The various factors such as drug surface properties, size and shape, targeting, and circulatory time mainly regulate EPR-based drug delivery directly or indirectly. However, there are certain limitations associated with the EPR effect, e.g., tumor microenvironment and heterogeneity that varies in types of tumors with the same origin, models, and patients [26,27].

The tumor microenvironment plays a critical role in designing and developing nanoparticles or associated carriers [28]. Nanoparticles embedded with scaffolds have the advantage of producing a synergistic anticancer therapeutic effect by carrying multiple drugs as a single system in addition to overcoming the challenges of delivering hydrophobic drugs [28,29,30]. Furthermore, by improving immunotherapy, nanodrug delivery carriers such as nanoparticles have been demonstrated to be potential candidates for reversing the immunosuppressive microenvironment of tumors [31]. Altogether, for anticancer therapy, there are several kinds of approaches for developing nanoparticles and for the materials used, which include polymers, lipids, organics, inorganics, ceramics, etc., and are presently validated for their potential anticancer therapeutic effects [32,33]. The use of drug toxicity as an antitumor chemotherapeutic at tumor sites has shown the potential to kill tumor cells and significantly regulates tumors and avoids recurrence. However, such approaches have led to the killing of normal cells in the surrounding tissues, leading to systemic side effects [34]. Scaffolds, as a targeted or localized toxicity-inducing implantable/injectable delivery platform for antitumor effects, are emerging as a promising approach [35]. These injectable scaffolds can deliver drugs in a minimally invasive way or implant at the site, also showing the ability to release multiple drugs in a controlled and sustained manner, leading to a multifunctional therapeutic effect [36]. However, there are certain major limitations associated with intratumoral administration, which include biodegradability, immunogenicity, ensuring they are highly efficient and responsive to tumors, the spatiotemporal release of anticancer drugs from the depot, the need for a trained person to administer them, etc., that still require considerable attention [37].

Furthermore, different types of nano/micro-drug delivery systems involving 3D scaffolds have been developed throughout the decades that differ from each other in terms of composition, crosslinker, ionic charge, pore size, release kinetics, and method of preparation. This paper summarizes the materials used for scaffold preparation and the development of smart and stimulus-responsive scaffolds that have been proposed as next-generation chemotherapeutics nano-delivery depots to address the problems associated with traditional chemotherapy’s ability to deliver drugs into tumor tissues and improve local cancer therapy.

## 2. Biomaterials for 3D Scaffolds

**Figure 1:**
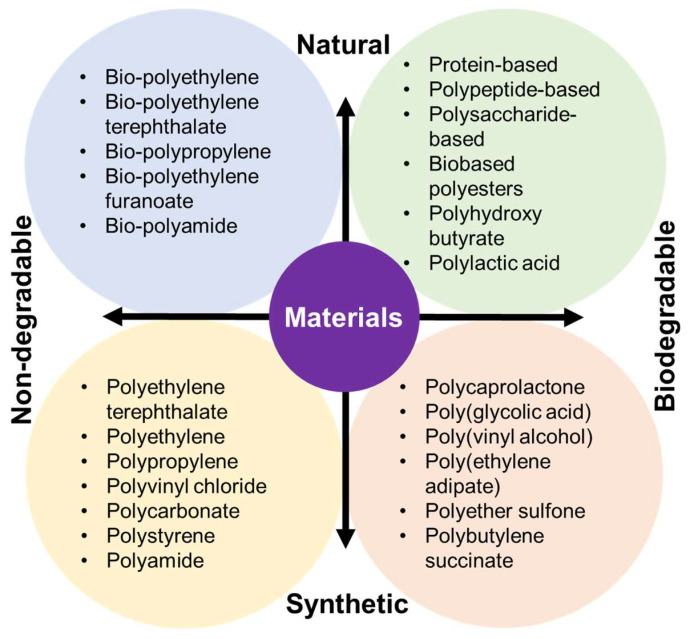
Classification of biomaterials used to prepare 3D scaffolds according to their sources and biodegradability.

A series of FDA-approved biocompatible polymers have already been established to develop a variety of 3D scaffolds to treat cancer recurrence [38]. However, the type of tumor microenvironment, the type of metastasis, the cellular interaction, and the type of chemo drugs and immunotherapeutics must be carefully considered to select a polymer [39]. Depending on each unit’s structure–property relationship (SPR), composition, and functional group, a polymer can be degradable or nondegradable, as well as hydrophilic or hydrophobic, thereby facilitating diverse anticancer activities. 3D scaffolds are of interest for cancer immunotherapy due to their unique characteristics such as high porosity, high surface–volume ratio, controllable mechanical properties, ability to interact with cytotoxic T lymphocytes, etc. [40]. Figure 1 illustrates the classification of polymers used to make different scaffolds according to their sources (e.g., natural or synthetic) and their degradation profiles.

### 2.1. Natural Biomaterials

Natural polymers have been used over the past few decades to develop a variety of scaffolds for cancer treatment [41]. They can be classified into four categories according to their structure and nature: polypeptide-based, protein-based, polysaccharide-based, and polynucleotide-based natural polymers [42]. Natural polymers consist of long chains, and bio-functional units represent their activity, hemocompatibility, three-dimensional geometry, and underlying internal structure. Although natural polymers have advantages in fabricating anticancer scaffolds, they also have disadvantages, such as being prone to bacterial or fungal contamination, unwanted immunogenicity, and a weak degradation profile [43]. Hence, considering the type, stage, and microenvironment of the tumor, these polymers can be modified to have desired properties in fabricating the scaffold for the indispensable anticancer effects. The SPRs, advantages, and disadvantages of different natural materials are shown in Table 1.

### 2.2. Synthetic Biomaterials

Synthetic biomaterials are manufactured polymers derived mostly from petroleum oils and macromolecules in nature [58]. Such biomaterials are classified into three categories, namely thermoplastic, elastomeric, and synthetic polymers. A synthetic polymer can give rise to another synthetic polymer by changing its main or side chain [59]. The backbone of a synthetic material is primarily made up of carbon chains but may also contain hydrogen and/or oxygen bonds [60]. An exception to this pattern among synthetic materials is silicon, which has no carbon atoms and is therefore called an inorganic substance. Various synthetic materials are used to make anticancer scaffolds because of their advantages over natural polymers, such as high functionality, tunable properties, physicochemical stability, and mechanical strength. However, it is infrequent for a single synthetic material to have all of these properties. Table 2 presents a list of synthetic polymers as well as their advantages and disadvantages.

## 3. 3D Scaffolds for Chemotherapeutic Delivery and Cancer Treatment

The choice of scaffolds and chemotherapeutics depends on the types of cancer being treated. Furthermore, the drug-loading method is chosen according to the dosage form, types of polymers, and the site of application. In Table 3, we have tabulated the therapeutic results of chemotherapeutic-loaded scaffolds for several types of cancer.

### 3.1. Smart Scaffolds

**Figure 2:**
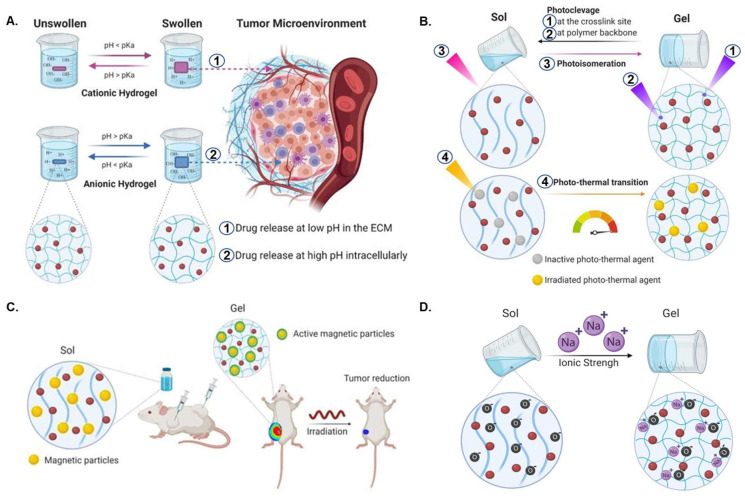
(**A**) Schematic illustration of pH-responsive scaffolds. (1 and 2). Site-specific chemotherapeutic releases at low and high pH, respectively. (**B**) Photo-induced cleavage (1 and 2), isomerization (3), and heat change (4) in chemotherapy drug-loaded light-sensitive scaffolds. (**C**) Magnetic scaffolds for tumor suppression. (**D**) Ion force-triggered scaffolds. Red-colored balls represent anticancer drugs. Reproduced with permission [8]. Copyright 2021, MDPI.

The arrangement of the fibers within the scaffold, the porosity, and the materials can be adjusted based on the potential use of the scaffold. A series of smart scaffolds such as pH-sensitive, photo-responsive, ionic force-sensitive, magnet-responsive, and dual-padlock scaffolds have been developed to enhance the therapeutic efficacy of cancer therapy and reduce unwanted side effects (Figure 2). These smart scaffolds can reduce drug toxicity, control the time of gelation in tumor tissue and the time-dependent release of chemotherapeutics, and improve bioavailability.

The extracellular matrix of the tumor microenvironment is an acidic state, whereas an alkaline state characterizes the intracellular environment [85]. These unique features of tumor microenvironments allow researchers to develop pH-sensitive scaffolds that can selectively deliver drugs to tumor tissue and thereby protect healthy cells from unwanted death [85]. The pH-sensitive scaffolds are classified into two distinct groups: the cationic and anionic scaffolds [86]. The release profiles of these materials depend on the polymer concentration, biodegradation, and swelling ratio of the scaffolds in the tumor microenvironment [87]. The behavior of pH-sensitive polymeric hydrogels can be modulated by changing the ionization degree, concentration, charge, etc. [88]. When the pH is lower than the pKa value, cationic scaffolds become protonated, leading to the release of the drugs at a lower pH. In contrast, anionic scaffolds release drugs when the pH is higher than the pKa value. Because the pH of the extracellular matrix of tumor tissues is low and the pH of the intracellular site is high, cationic scaffolds can be used to deliver chemotherapeutics at the extracellular milieu, and anionic scaffolds can be used to selectively deliver drugs into the cytoplasm (Figure 2A). This strategy significantly increases the synergistic effects of cancer treatment.

Raza et al. developed a pH-responsive, injectable hydrogel for the localized intratumoral delivery of paclitaxel using the FER-8 peptide. The pH-sensitive FER-8 peptide hydrogel was well characterized in vitro and validated for its targeting and antitumor effect in H22 tumor-bearing mice. The in vivo results demonstrated its pH-responsive controlled release of paclitaxel and substantial efficacy in inhibiting tumor growth [89]. Cui and co-workers developed doxorubicin (DOX) and ibuprofen (IBU)-containing mesoporous silica particles (MSNs) as a dual drug delivery system and incorporated the MSNs into a poly(L-lactide) electrospun scaffold. The DOX along with sodium bicarbonate was at the core of the MSNs for long-term release in response to pH, while on the outer side IBU was released rapidly. The results revealed significant necrosis, apoptosis, and prolonged mouse survival when the MSNs were implanted over 10 weeks in a liver tumor-bearing nude mouse [90]. Similarly, multiple studies have illustrated targeted anticancer effects involving approaches with anticancer drug delivery carriers based on injectable, pH-responsive hydrogels such as alginate polydopamine hydrogel [91], chitosan/(poly(*N*-isopropyl acrylamide-co-itaconic acid) hydrogel [77], F127–CHO (FC)-PPR-CMC hydrogel [92], and gelatin-based Gel-ADH/diBA-PEG/LAP@DOX hydrogels [85]. However such types of pH-sensitive scaffolds will be ineffective for cancer treatment (e.g., brain tumors) when the pH ranges from 6 to 7.2, and in such cases, selective pH-triggered scaffolds, separately or as a combinatory approach, can show potential and desired anticancer effects [93]. Bai et al. grafted graphene oxide (GO) onto a copolymer of polyacrylic acid-g-polylactic acid (PAA-g-PLLA) and fabricated a stimuli-responsive scaffold along with polycaprolactone (PCL) gambogic acid (GA). The scaffolds demonstrated selective tumor response and significant accumulation of GO/GA among in vitro breast tumor cells (MCF-7 cells) at pH 6.8 in contrast to normal cells (MCF-10a cells) at pH 7.4. The study also elucidated that the synergistic approach of using pH-responsive photo-thermal conversion was more effective than independent treatments. Finally, the in vivo results demonstrated remarkable tumor inhibition (99% in 21 days) through tumor tissue disintegration, degeneration, and tumor suppression in treatment with GO-GA scaffolds under photo-thermal treatment in comparison to the control, GO-GA scaffold, or NIR irradiation only [94].

Light-sensitive scaffolds are activated upon exposure to external stimulus in the form of light, and the scaffold response can be reversible or irreversible, depending upon the type of material and composition [95,96]. These photodegradable scaffolds can be prepared in four ways: cross-linking cleavage, backbone cleavage, photoisomerization, or photothermal transition (Figure 2B). The desired drug release at the tumor site mainly depends on the gelation, swelling ratio, depolymerization, and other physicochemical properties of the scaffold and can be achieved by tuning photo-stimulus properties such as light intensity, light dose, wavelength, exposure time, etc. [97,98]. The failure of light to penetrate deep tissues has limited the application of phototherapy [99]. To rectify this limitation, strategies of using phototherapy and other therapeutic approaches in combination can substantially improve its treatment potential in several types of cancers [99]. Thus, the extensive ability of advanced photodynamic therapy to penetrate deep tissues and impart spatiotemporal precision aids further in establishing it as a candidate of interest for localized cancer therapy [99,100]. Jin et al. in one such study developed an injectable hydrogel for tumor inhibition using a combinatory therapeutic approach of phototherapy and chemotherapy [101]. The study involves the development of an injectable hydrogel consisting of a genetically modified polypeptide (PC_10_A) and hollow gold nanoshells (HAuNS). The results demonstrated the sequential release of DOX from hybrid PC_10_A/DOX/HAuNS nanogels embedded in PC_10_A hydrogel, and significant tumor inhibition was observed in in vitro and in vivo tumor model experiments by using photo-chemotherapy compared to using the treatments separately. Wang et al. recently fabricated a photosensitive interpenetrating hydrogel network from chitosan and polyacrylic acid loaded with 5-aminolevulinic acid (ALA) for the treatment of oral potentially malignant disorders (OPMDs). The adhesive photo-responsive hydrogel revealed an enhanced effect in vitro and in a hamster oral carcinogenesis model [102]. Photo-crosslinked GelMA-based 3D printed scaffolds loaded with prodrug Pt(IV) as an initiator were fabricated for tumor treatment in postoperative procedures [103]. Microwave-responsive graphene scaffolds are of interest regarding the delivery of hydrophobic chemotherapy drugs at a lower frequency than hydrogel microwave-responsive scaffolds. The hybrid graphene–diaminotriazine scaffold can deliver hydrophobic drugs with the stimulus of microwaves at 915 MHz. This was found to be further enhanced by acidic environments as a result of the diaminotriazine (DAT) functionality. Graphene reduces the risk of overheating the tissue by dissipating the heat generated from microwaves [104]. The microwave-responsive scaffold is of interest for chemotherapy drug delivery because it can facilitate the delivery of hydrophobic drugs that otherwise may not be as clinically useful.

In addition to pH- and light-responsive scaffolds, magnetic-sensitive scaffolds are also very effective in treating cancer. This type of material is made by incorporating magnetic nanoparticles during the preparation of a scaffold. On applying a magnetic field, the scaffold undergoes hysteresis loss, causing temperature increase by the conversion of magnetic energy into a heat energy [105]. Magnetic-sensitive scaffolds containing iron oxide nanoparticles vibrate when they are exposed to alternating magnetic fields, which causes the temperature inside the cells to rise, resulting in thermal-ablation mechanism-mediated cellular death (Figure 2C) [8]. The temperature can be regulated by modulating various magnetic parameters, such as frequency and strength of the field, magnetic particle size, concentration, and other properties of particles [105]. Nevertheless, it is still under discussion that this temperature increase can promote cell damage directly, instead of only serving as a co-adjuvant of other therapies. Furthermore, the scaffolds begin to swell due to thermal ablation, leading to the release of chemotherapeutics [106]. Because the cell’s internal components come out at the time of cell death [107], it can increase the inflammatory response in the body, which is the main drawback of this treatment method. However, tumor cells release pro-inflammatory molecules to grow, and they are important in the metastatic process. Hence, partial necrosis of the tumor can have the opposite effect and trigger even greater growth. Magnetic-reactive scaffolds combined with light radiation therapy may play a synergistic role in tumor reduction [108]

Ion force-triggered scaffolds undergo structural changes in response to cations. Zwitterionic polymers such as PAA, PIA, CMD, and XIP are commonly used to make such scaffolds [109]. It has been reported that the ionic force of materials is proportional to the release profile of the ionic scaffold (Figure 2D). A high ionic concentration can break down electrostatic interactions between scaffolds and doxorubicin and cause a burst release of doxorubicin in the target region [110].

Dual-functioning scaffolds hold two roles: first as a drug delivery scaffold, and then as a tissue-regeneration scaffold. The damage caused by cancer can also result in the need for reconstructive surgery, which includes the implantation of a polymeric scaffold. As mentioned previously, systemic chemotherapy is not ideal for localized tumors, as it causes many unwanted adverse effects and trauma to the patient. A dual-functioning scaffold can be placed at the time of initial surgery, where it will sustain the release of chemotherapy drugs to the localized area. After the drug is released, the scaffold will be left as a scaffold for tissue regeneration. The architecture is suitable for cell migration, proliferation, and adhesion [36].

Despite the existence of many smart scaffolds, dual-padlock scaffolds are considered the most promising scaffold-based treatment strategies for treating cancer [77]. In this system, the scaffolds are designed so that a single scaffold can work both in temperature- and pH-sensitive manners. Such scaffolds are usually injectable and form a gel after administration in the tumor tissue at body temperature. Subsequently, they kill cancer cells by selectively delivering different chemotherapeutics to intracellular and extracellular sites.

Under a laser light with a specific wavelength, the crosslinkers are activated and create internal pressure, which induces gelation-mediated contraction of the scaffold’s structure [15]. Because of these advantages, researchers are showing a tendency to use these types of scaffolds for tumor-starvation therapy to treat metastasis and recurrence. Starvation therapy can destroy malignant tumors by shutting off the supply of nutrients and oxygen to cancer cells [111]. A recent study focused on establishing an appetite therapy using a shape-shrinking hydrogel that can shrink after exposure to 808 nm laser irradiation and create internal pressure to constrict blood vessels, which blocks blood flow and thereby treats pancreatic and breast cancers. This therapy has also had pre-clinical success regarding lung cancer metastasis and recurrence [15].

Shape-compressing microspheres can also be used to deliver drugs to the site of treatment. Du et al. report a shape-memory scaffold composed of poly(lactide-co-glycolide) microspheres. The microspheres were loaded with vancomycin in this study and then compressed to form the scaffold. Once placed, the expanded microsphere scaffold released the vancomycin at a lower rate than that of microspheres alone [112]. This design has the potential for the delivery of antibiotics in confined spaces, such as sites of bone regeneration. This same concept can be applied to cancer treatment when resection of the entirety of the tumor is unfavorable due to other local structures.

### 3.2. Expandable Scaffolds

Recent advances in converting two-dimensional electrospun nanofibers into three-dimensional hierarchical structures have attracted attention for simultaneously delivering multiple drugs [113]. Using gas-foaming technology, it is now possible to create different pore sizes in the same scaffold, which allows the loading of different anticancer drugs into different scaffold pores [114]. Previous studies have shown a specific release profile for a specific drug delivery platform [115]. Although it was easy to load multiple drugs into the same scaffold, it was impossible for them to have different release profiles within a specific drug delivery platform. However, expandable scaffolds show different release profiles for different drugs even though they are included in a single scaffold.

Zhang et al. proposed a 3D-printed scaffold vaccine for the delivery of immunoregulators. This scaffold is porous, which mimics the function of lymphoid organs. The scaffold is able to recruit immune cells to the site while delivering immunoregulating drugs to target cancer. Both humoral and cellular immune responses are shown to be strengthened using this approach. The 3D-printed scaffold showed better cell influx and recruitment than the existing hydrogel vaccines [116]. Additionally, the scaffold can be customized through 3D printing and implanted in a surgical setting after resection to prevent future metastasis.

### 3.3. Microneedle Patch

**Figure 3:**
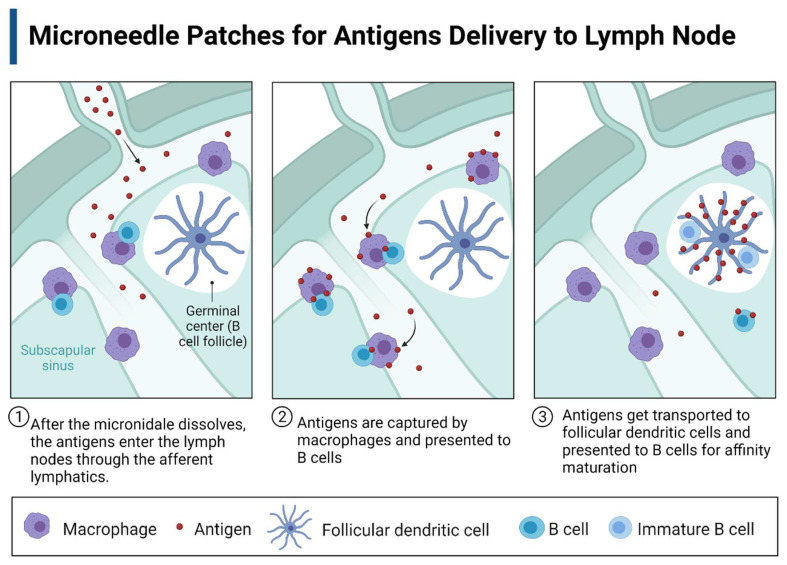
Microneedle scaffold-based tumor-specific antigen delivery to the lymph node. (1–3) B cells encounter antigen and process it.

Microneedle patches alleviate the complications associated with the traditional injection-based delivery of chemotherapy drugs such as first-pass metabolism, toxic effects on normal cells, unwanted side effects, inflammation, and discomfort at the injection site [117]. In addition, their high biocompatibility, biodegradability, degradation-based drug release properties, and ability to mimic the extracellular matrix make them a suitable platform for the delivery of anticancer drugs [118]. For example, a sodium hyaluronate-based microneedle can be used to overcome the weak penetrability of the 5-aminolevulinic acid [119]. This scaffold enhances drug delivery efficiency and reduces side effects compared to IV injections. Also, it has been reported that microneedle patches can efficiently inhibit cancer cell proliferation and metastasis by delivering a chemotherapy drug called doxorubicin to the lymph nodes [120]. After the microneedles are inserted into the skin, doxorubicin is secreted by dissolving the microneedles and accumulates in the subcutaneous lymph nodes. This study suggests that microneedle patches may be used to deliver tumor-specific antigens to subcutaneous lymph nodes to treat melanoma (Figure 3). Antigens will enter the lymph nodes by dissolving microneedles through afferent lymphatics in the subscapular sinus, and then the antigens will be captured by the subscapular sinus macrophages and presented to the B cells. Eventually, the antigens will be transferred to the follicular dendritic cells and presented to the B cells for affinity maturation.

The skin is a highly immunogenic organ. The barrier function of the skin necessitates immune cell presence, as it is the first layer of defense against injury and invasion. Malignancy of skin cancers is most common in humans, and treatment with immunotherapies by targeting checkpoint inhibitors in recent years has shown promising potential [121]. Furthermore, tumors are able to evade the immune system through upregulation of programmed cell death ligand 1 (PD-L1) on tumor cells, which binds to programmed cell death protein 1 (PD-1) on T cells, keeping them in an inactive state [122]. Dermal delivery of lipid-coated nanoparticles containing anti-PD-1 drugs increases T cell activation and immune response to tumor cells. Additionally, this can be used in combination with chemotherapy drugs for a synergistic effect on surface tumors.

Wang et al. showed an enhanced immune response in a melanoma mouse model using anti-PD-1 (aPD1) immune therapy. The study demonstrated controlled delivery of aPD1 by a microneedle patch based on hyaluronic acid incorporated with dextran nanoparticles. The aPD1 and glucose oxidase were embedded in pH-responsive dextran nanoparticles. Under an acidic microenvironment, the microneedle patch undergoes self-degradation to release the drug [123]. However, the clinical translation of checkpoint inhibitor-based immune therapy is limited by certain limitations such as acute systemic side effects, reduced objective response rate, etc. Chen et al. designed hollow-structured microneedles integrated with checkpoint inhibitor-based immune therapy for the delivery of anti-programmed death-ligand 1 antibody. The immune therapy was mediated by cold atmospheric plasma to kill tumor cells by transdermal release facilitated by hollow microneedles. The study elucidated promising tumor growth inhibition as well as improved survival time of tumor-bearing mice [124]. In another study, Lan et al. explored a combinatory approach by using immune therapies and chemotherapies for effective cancer treatment through a synergistic effect. The therapeutic agents (anti-PD-1/cisplatin) were encapsulated into pH-responsive nanoparticles and were loaded into microneedle patches [122]. Kim et al. described remarkable tumor inhibition prompted via cellular and antigen-specific humoral immunity induced by the simultaneous delivery of hydrophilic tumor antigen and hydrophobic resiquimod (R848) by intradermal microneedles [125].

Microneedle design can be adjusted for specific release profiles based on therapeutic use. For example, polymers can be chosen to correlate with the desired release time for either a quick release or sustained release of a drug [126]. Additionally, microneedles can be fabricated using materials with other anticancer effects. Separable microneedles including doxorubicin and indocyanine green were used to synergistically utilize chemotherapy and photothermal therapy to treat superficial tumors. The structure of the microneedles is arrow-like, with a dissolving base made of poly(vinyl alcohol) (PVA) and polyvinyl pyrrolidone, as well as an arrow tip that contains the active drugs. When inserted into the skin, the poly(vinyl alcohol) and polyvinyl pyrrolidone will dissolve, leaving the tip of the arrow embedded into the skin. Phototherapy with near-infrared light can then be used to convert the indocyanine green, resulting in physical heat and tumor cell death [127]. The effect of phototherapy is synergistic with the release of doxorubicin, resulting in increased penetration and treatment of the superficial tumor. Phototherapy shows great promise for the treatment of superficial tumors, as it can be controlled through the placement of photothermal conversion agents. The tumor cells can be targeted for the uptake of these agents, thereby leaving the healthy cells virtually untouched in the procedure.

### 3.4. Microspheres

**Figure 4:**
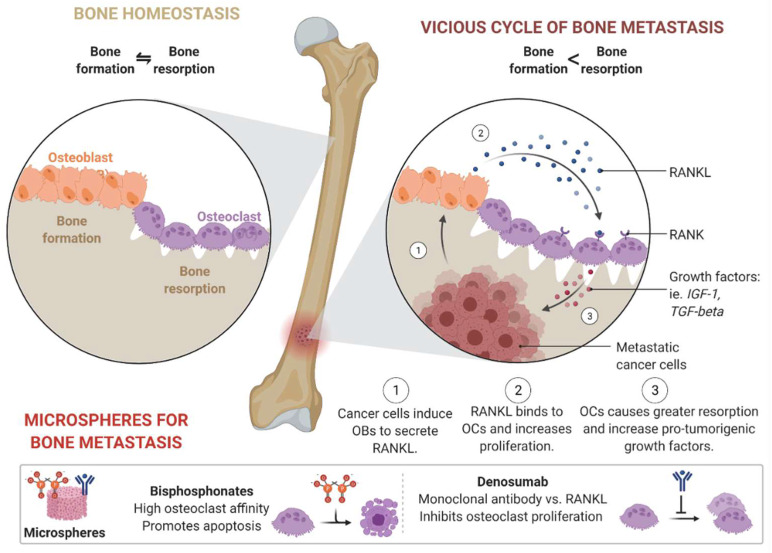
New techniques for the treatment of bone metastasis using the microsphere as a controlled release depot.

Microspheres are small porous particles with an excellent ability to load and release drugs in a controlled manner [128]. Injectable microspheres are fabricated by co-axial electrospinning and can be used for minimally invasive delivery of drugs inside bone-like rigid structures without surgery. Due to the excellent microarchitecture of the microsphere, researchers nowadays tend to use injectable microspheres to treat bone metastasis [129]. In bone metastasis, cancer cells induce osteoblast expression to release an apoptotic regulatory gene (RANKL) so that the RANKL may bind to the osteoclast and increase proliferation. As a result, osteoclasts cause more resorption and induce pro-tumorigenic growth factors (Figure 4). Microspheres can be used to treat bone metastasis by promoting apoptosis, inhibiting osteoclast proliferation, or combining both techniques. Bisphosphonates and denosumab are drugs used to treat bone metastasis [130,131]. However, the main problem with these two drugs is their rapid deterioration and repetitive administration, making it difficult to perform treatment with traditional methods. Both drugs can be easily loaded into a microsphere during preparation, and their release can be controlled due to the presence of microchannels inside the microsphere. The long-term stability of this type of microsphere may provide one-time treatment options for patients with bone metastasis. Because bisphosphonates have an affinity for osteoclasts, they promote apoptosis after release from the microsphere. At the same time, a monoclonal antibody called denosumab inhibits osteoclast proliferation. Because both drugs can be continuously released from the microsphere, there would be no need to repeat the administration.

Microspheres can be fabricated using a variety of polymers, which determine stability and biodegradation profiles. Ni et al. report a poly(lactide-co-glycolide) (PLGA) microsphere that displays high photothermal conversion efficiency while simultaneously delivering cytotoxic doxorubicin to tumor cells. When the microspheres are injected into the tumor, they induce local hyperthermia. Damage from physical heat and toxic effects of the chemotherapy work in synergy to treat the tumor while avoiding systemic adverse effects [132].

## 4. Nanotechnology-Based Treatment Approach

**Figure 5:**
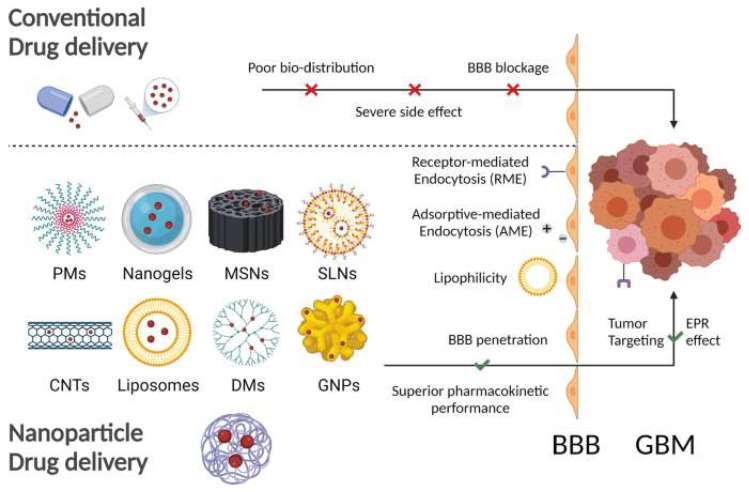
Representation of different nanoparticles such as polymer nanoparticles (PMs), nanogel, mesoporous silica nanoparticles (MSNs), solid lipid nanoparticles (SLNs), carbon nanotubes (CNTs), liposomes, dendrimers (DMs), and gold nanoparticles (GNPs) and how they can overcome the limitations of traditional anticancer treatment [139].

In recent years, anticancer drugs such as alkylating and alkylating-like agents, antimetabolites, antitumor, antibiotics, etc. [133], have been considered the most preferred therapeutic option in cancer treatment. Aiming for targeted therapy and side effect reduction, nanotechnology-based cancer therapies have received increased attention as the most potent approach in the field of oncology [134]. Many papers in the literature have described in detail how nanotechnology can be a potential anticancer treatment strategy [135,136,137,138]. We will give a brief summary covering certain major types of approaches for this platform (Figure 5).

### 4.1. Nanoparticles (NPs)

Nanoparticles (NPs) have plasticity in size control, composition, and surface modification for effective delivery to the targeted site, leading to improved therapeutic efficacy and side effect minimization. Over the past few decades, a wide range of NPs have been proposed, such as magnetic nanoparticles (MNPs), inorganic nanoparticles (INPs), polymer nanoparticles (PNPs), lipid nanoparticles (LNPs), etc. MNPs are NPs that have magnetic susceptibility which can be controlled by an external magnetic field. The most used material in MNPs is the iron oxide nanoparticle, together with magnetite (Fe_3_O_4_) and maghemite (γ-Fe_2_O_3_) [140]. MNPs are considered the most widely used cancer imaging tool for cancer diagnosis due to the high spatial resolution and tomographic capabilities of magnetic resonance imaging (MRI) [141]. Wang and co-workers produced sur-MNPs by the conjugation of chitosan-coated MNPs and survivin antisense oligonucleotides (ASON) and found that the MNPs functionalized with ASON led to targeted localization in pancreatic tumors. They observed that surviving tagged NPs could be used in the diagnosis of pancreatic tumors by MRI [142]. Because MNPs can convert electromagnetic energy into heat, these NPs can kill tumor cells by heating them to the threshold level of apoptosis [143]. A successful in vitro and in vivo study where the MNPs destroyed glioma cells was conducted by Cheng and colleagues [144]. PNPs can also execute cancer therapeutic roles successfully by different mechanisms. For PNP formulation, both natural and synthetic polymers can be used. The natural polymer chitosan and its derivatives are found to be efficient in anticancer drug delivery and effective in cervical cancer inhibition [145]. A targeted moiety of hyaluronic acid was tagged on the surface of PLGA nanoparticles by Cerqueira and co-workers, and a targeted delivery of paclitaxel (PTX) was observed in triple-negative breast cancer [146,147]. INPs such as gold NPs, zinc NPs, silver NPs, iron NPs, and very recently novel INPs with nanodiamonds and graphene have been explored in the context of cancer treatment [148,149]. Gold NPs functionalized with tyrosine kinase inhibitor have been explored as a delivery vehicle and found to be effective in the inhibition of dual tyrosine kinase in metastatic breast cancer treatment. In a study by Tsai and associates, it was observed that gold NPs coated with epigallocatechin gallate (EGCG)/gallate has the ability to enhance the efficacy of doxorubicin delivery and inhibit PC-3 cancer cell proliferation in prostate cancer [150,151]. Silver NPs have the dual function of anticancer therapy as well as cancer cell imaging. Boca-Farcau and colleagues showed that silver NPs along with surface conjugation of folic acid and labeled with a p-aminothiophenol Raman reporter molecule can be used as a targeted cancer cell treatment. Another study demonstrated the cancer cell diagnosis and imaging capacity of silver–gold nanorods [152,153,154]. A lipid-based nanosystem was first introduced into the market by the Dior brand in 1986 [155]. However, it gained popularity after COVID-19 emerged like never before. LNPs can overcome several obstacles encountered in other nanoparticle-based anticancer drug delivery systems such as tissue toxicity, poor cellular uptake, particle aggregation, etc. Hence, LNPs are identified as a promising nanoparticle-based delivery method. This system can co-encapsulate a treatment moiety and imaging agent, providing both therapeutic and diagnostic functionalities [156]. In 2021, Prasad and co-workers developed lipid theranostics by incorporating gold nanoparticles as an imaging tool along with a chemotherapeutic agent. They also performed surface modification by tagging folic acid for making a targeted delivery. They demonstrated that their particle showed enhanced cellular internalization at the target site [157] Another research study proposed LNP cancer theranostics in lung carcinoma, where they encapsulated paclitaxel and siRNA as a therapeutic agent and quantum dots (QD) as a diagnostic agent. This study found a synergistic effect of siRNA and paclitaxel in effective nanoparticle tracking as well [158]. A combination of lipid and polymer yielded a lipid–polymer hybrid, which is a next-generation lipid nanocarrier. This system has a core of polymers which is enclosed by a shell of the lipid bilayer. In recent times a research group developed hybrid lipid–polymer NPs having an ultrasound contrast agent and a prodrug. This type of NP demonstrated reduced toxicity and excellent anticancer activity in ovarian carcinoma [159].

### 4.2. Nanospheres

Nanospheres are also emerging for the delivery of chemotherapeutic drugs to tumor cells. Biodegradable polymers can be used for the fabrication of nanospheres, resulting in a safe, degradable, and effective vector. Polylactic acid (PLA), PGLA, and poly(ε-caprolactone) PCL can be used as polymers for the nanospheres. Cancer creates a microenvironment of a dense extracellular matrix with increased angiogenesis. The newly formed vasculature within the tumor is immature and therefore is less secure, facilitating leakiness. For classic chemotherapeutics, this acts as a size-selective barrier. Nanospheres are small enough to exit the blood and accumulate in the tumor. The microenvironment of the tumor also suppresses normal lymphatic drainage, so the nanospheres are able to remain in the tissue for longer periods of time [160].

The blood–brain barrier is another highly selective mechanism preventing the accumulation of some chemotherapeutic drugs for the treatment of central nervous system tumors. The small size of nanoparticles combats this barrier, allowing for the treatment of central nervous system cancers with chemotherapy while avoiding invasive procedures such as intraventricular administration [161]. The uptake of nanoparticles can be enhanced using conjugation to ligands that improve uptake across the barrier. The use of nanospheres is not limited to drug delivery; they can also be used without a drug for anticancer effects. Zinc oxide nanoparticles have been used to generate reactive oxygen species when used with a UV radiation [100]. This can help to reduce the effect of drug resistance on cancer treatments, allowing for the treatment of multi-drug-resistant tumors.

### 4.3. Exosomes

Exosomes are widely used physiologically for the transport of cargo, intracellular signaling, and the maintenance of homeostasis. Exosomes are very small, usually around 30–100 nm in diameter [162]. The contents are enclosed in a phospholipid bilayer, as they are released from the invagination of the extracellular membrane and exocytosis. The membrane also contains proteins, lipids, and nucleic acids that are typically present on the surface of the cell of origin. Encased within the exosome are proteins, mRNAs, miRNAs, and enzymes that come from the cell of origin. The structure of the exosome allows for the delivery of contents to another cell through a variety of pathways, such as clathrin-mediated endocytosis, lipid raft-mediated endocytosis, heparin sulfate proteoglycan dependent endocytosis, phagocytosis, or direct fusion [163]. Physiologically, this is important for cell survival, immune response, and cell signaling. Exosomes also have a role in the pathology of cancer, as they are released by tumor cells, resulting in migration and metastasis. The release of exosomes by tumor cells also can adjust the tumor microenvironment through extracellular matrix remodeling, immune evasion, drug resistance, and angiogenesis [164]. In addition, the exosome has a cellular feature at its membrane which makes it more biocompatible and reduces tissue toxicity and immunogenicity.

Exosomes are an excellent area for chemotherapy delivery, as they can be used to safely deliver cytotoxic drugs to cancer cells [163]. The structure of the exosome is stable for travel throughout the blood. The nature of the exosome also allows for the delivery of drugs across the blood–brain barrier, which is quite difficult for many chemotherapy drugs [165]. Utilizing brain-specific surface markers can also increase the uptake of exosomes across the blood–brain barrier [163]. The use of exosomes can reduce the systemic toxicity of chemotherapy by selectively targeting tumor cells. Doxorubicin-loaded exosomes were studied for chemotherapy delivery and showed decreased cardiotoxicity compared to other drug delivery methods [166]. As the exosomes are endogenous structures, there is low immunogenicity associated with delivery.

Delivery of 5-fluorouracil was also studied using exosomes in conjunction with microRNA inhibitors in order to combat drug resistance in colorectal cancer [167]. Multiple drug resistance (MDR) is one of the major challenges in cancer. Exosome-mediated anticancer drug delivery is found to be effective in overcoming MDR in cancer treatment. One study found that the antimitotic agent paclitaxel (PTX) loaded into exosomes is effective in overcoming MDR. It also showed higher cellular uptake and had an IC50 lower than that of free PTX. Therefore, decreased tissue toxicity was observed [168]. Another study by Srivastava and associates demonstrated that delivery of exosomes loaded with doxorubicin conjugated with gold nanoparticles (GNPs) showed preferred toxicity to lung cancer cells over healthy cells [169].

### 4.4. Nanogels

Nanogel systems are nanomaterials having hydrophilic groups, for instance, hydroxyl (-OH), carboxyl (-COOH), amino (-NH_2_), amide carboxyl (-COOH), and sulfonic (-SO_3_H) groups. They have a similar internal structure to hydrogel or microgel except for the size of the particles being in the nanoparticle range [170,171]. Huang and his group produced doxorubicin (DOX)-loaded amphiphilic nanocarriers which showed better cellular uptake as well as better targeting efficiency [172]. Oh and associates synthesized a pH-responsive drug carrier using glycol chitosan (GCS) and loaded it with DOX. This system can recognize the pH of the tumor and hence showed better in vivo therapeutic activity of the drug targeting the tumor [173].

Though NPs and exosomes can overcome several drawbacks associated with conventional cancer therapies, there are still some challenges that need to be addressed. For example, the targeting agent can change some properties of the NPs such as particle size, stability, solubility, pharmacokinetics, etc. Some NPs, for instance, poly (lactic-co-glycolic acid) (PLGA), degrade rapidly and have low circulation time in tissues which is not enough for sustained drug delivery [174]. On the contrary, the carbon nanotube can exist in the body for a larger time period, sometimes even for months or years, which can cause potential toxicity [175]. Limited vascular permeability in some types of tumors hinders the tumor penetration capacity of the NPs which results in suboptimal drug delivery. Moreover, slow drug release can affect the bioavailability of the drug [176]. Nanoparticles are mostly injectables. Various publications reported post-injection accumulation of nanoparticles at the injection site. In addition, frequent dosing by injection can cause infection at the site of injection which results in poor patient compliance. Exosomes have an advantage over NPs in several aspects but still face difficulty in large-scale production. Hence, it is still a challenge to perform clinical trials with exosomes [177].

These problems associated with anticancer drug delivery by NPs could be addressed by incorporating the NPs into different types of natural or synthetic polymer scaffolds or microneedle patches. The injectable scaffolds or microneedle patches would be minimally invasive. This would reduce the chances of infection at the administration site. Furthermore, the slow release of drugs from the scaffold would also resolve the injection-site particle accumulation phenomenon. Anticancer agents which stay longer in circulation can lead to tissue toxicity. In this case, the drugs loaded into NPs can be incorporated into light-sensitive scaffolds which have a reversible activation upon light exposure. Thus, controlled drug release can be ensured to maintain a safe drug concentration in circulation. Again, a sustained drug release can also be assured by different scaffolds. In some cases, multiple anticancer therapies show a synergistic effect. However, incorporating different moieties into a single NP can interfere with the particle size, morphology, and release properties of the particle. To deal with this issue, different anticancer agents can be incorporated into different NPs, and then they can be loaded into a single scaffold. In this way, multiple anticancer therapies can be given simultaneously without them interfering with each other. The challenge of large-scale exosome production can also be overcome by adopting 3D scaffold devices. For example, by the application of 3D culture and tangential flow filtration (TFF), exosome production can be increased to a significant level [178].

Therefore, the conclusion of the above discussion is that different types of NPs, exosomes, nanogels, etc., have been proven to be an advanced form of therapeutic approach in cancer treatment. However, there are still some limitations associated with these treatment approaches which can be overcome if these NPs are incorporated with 3D scaffolds or microneedle patches. Because both NPs and polymeric scaffolds have their own advantages, having both of them in a single system can provide a synergistic effect. Table 4 summarizes the examples of scaffolds/hydrogels in clinical trials for the treatment of cancer.

There are several different types of NPs in clinical trials for cancer therapy. Among them, ABI-009 (NCT03817515) has already reached the market. In the following Table 5 we have mentioned some of them as examples.

## 5. Bacteriophage

Viruses have been studied for over 120 years. They cause infection by invading host cells and disrupting cellular functions [180]. Phages typically carry genetic material that allows them to replicate and carry out viral functions. Cancer therapy can utilize the age-old technology of viruses by loading chemotherapy drugs into phage nanoparticles [181]. Bacteriophages hold potential for drug delivery, as they are non-pathogenic to humans, and they can be genetically modified to fit the desired parameters [180]. The nanoscale size of phages allows for greater tumor permeability and evasion of clearance through the spleen, kidney, and liver [182]. There is also potential for delivery of chemotherapy to brain tumors through the crossing of the blood–brain barrier, which is a limitation of typical chemotherapeutic drugs [183]. The capsid of the bacteriophage can be altered through genetic insertion or chemical conjugation to specifically target ligands. Ashley et al. used bacteriophage MS2 to target hepatocellular carcinoma, delivering a cocktail of chemotherapeutics selectively to the tumor cells. The entire population of hepatocellular carcinoma cells was effectively killed using this delivery system, while the control cells were left viable, indicating successful specificity [184]. Bar et al. used antibody conjugation to control host selection, which allows for cancer targeting and selective delivery of cytotoxic drugs to a tumor, also leaving the healthy tissue untouched [185].

## 6. Conclusions and Perspectives

Considerable progress has been made in addressing the current limitations of scaffolds and improving their physical properties for chemotherapeutic delivery, but several drawbacks remain. Those challenges should be solved to improve clinical applications.

Premature gelation of scaffolds in the syringe can occur, which degrades the chemotherapy drug. Hence, the gelators and gelation process should be controlled. In addition, scaffolds that use crosslinkers to load chemotherapeutics are difficult to remove, even sometimes requiring further surgery that limits their application in the clinic. Researchers should focus on developing biodegradable and biocompatible crosslinkers to eliminate the potential risk of systemic toxicity. Enhancing the release period will be effective in cancer treatments and may allow scaffolds to replace hydrophobic systems for longtime release profiles. The development of scaffolds that can modulate the rate of chemotherapeutic delivery to the target cells can also be beneficial for cancer treatment that requires different doses of chemotherapy drugs over time. Scaffolds with time-dependent degradation profiles might be useful to address these challenges. Another potential problem of scaffold-based cancer therapy is the degradation and misfolding of sensitive therapeutics, such as small proteins, monoclonal antibodies, and nucleic acids inside the scaffolds. This is the main problem of in situ crosslinked scaffolds, in which the hydrophobic moiety of polymers reduces the bioactivity of the encapsulated chemotherapeutics. It is also necessary to develop biphasic scaffolds to deliver two types of chemotherapeutics at the application site simultaneously. The development of multiphasic scaffolds is of interest to enhance local chemotherapy. In addition, developing intelligent scaffolds should not be limited only to delivering chemotherapeutics. Future applications of scaffolds should include how to cure cancerous wounds in addition to chemotherapeutic delivery. Pore-size-dependent multiphasic scaffolds could be a promising area of interest that can control the release of different drugs at different stages of the cancerous wound; in addition, healthy host cells would be able to penetrate inside the scaffolds to heal damaged skin.

Advances in these challenges will efficiently improve the applicability of scaffold-based cancer treatments so that next-generation scaffolds can be successfully used to deliver chemotherapeutics at the desired rate and location. There is a wide range of applications for 3D scaffolds that is not covered in this review where options for improvement can be found.

## Figures and Tables

**Table 1:** Natural materials used to prepare 3D scaffolds.

Natural Materials and Their Chemical Structure	SPR	Merits	Demerits	References
Collagen 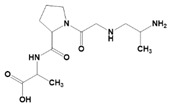	Hydrogen bonds hold the structure. Presence of glycine, proline, and hydroxyproline.	Biocompatible and biodegradable. Non-toxic. Less immunogenic. Extracellular matrix secretion.	Poor mechanical properties. Less stable.	[44]
Fibrinogen 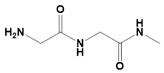	Presence primary and secondary amines in the structure. It consists of polypeptide chains.	High cellular uptake. Hemostatic properties. High cell adhesion properties. High surface-to-volume ratio.	Fast degradation. Poorly stable.	[41]
Gelatin [45,46] 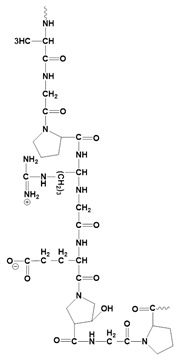	Consists of glycine, proline, and 4-hydroxyproline.	It can be used as a crosslinking agent. It helps to enhance the expansion ratios of other polymers. Excellent cell adhesion, proliferation, and differentiation properties. Less immunogenic. Biodegradable. Biocompatible.	Low stability.	[45,46]
Keratin	Presence of cysteine residues. Structural stability comes from intermediate filaments.	Excellent cell proliferation properties. Self-assemble. High cell viability. Controlled release properties. Time-dependent degradation profile.	Poor structural integrity at biological environment.	[47]
Starch 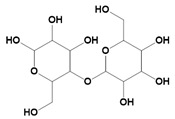	Consists of α-glycans. Carbohydrates.	Cytocompatibility. Excellent cell adhesion profiles. Highly hydrophilic Biodegradable. Suitable for photothermal therapy.	High water absorption ability. Poor mechanical properties. Difficult to chemical modification.	[48]
Chitosan 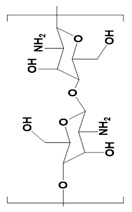	Linear polysaccharides. Beta-(1→4)-linked D-glucosamine	Highly porous structure. Hemostatic properties. High thermal stability. Inhibits liver metastasis. Inhibits growth factor-based proliferation of tumor cells.	Poor solubility in water. Susceptible to proteolytic enzymes.	[49,50]
Chitin 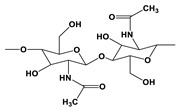	Presence of N-acetylglucosamine and N-glucosamine	It can be used for tissue repairing after breast cancer surgery. Non-toxic. Anti-inflammatory. Inhibits angiogenesis in tumors.	Poor stability. Poor solubility.	[51]
Agarose 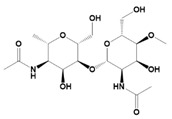	Agarobiose units are linked by hydrogen bonds.	Injectable in liquid form that later forms gel at body temperature. Excellent for cell delivery to target organs. It does not enhance immunogenicity. Biocompatible and biodegradable.	Non-degradable. Poor cell attachment.	[52,53]
Alginate 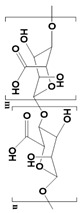	Different units of alginate have different properties. Presence of -COOH groups that can be chemically linked with anticancer drugs. Presence of guluronate units that inhibit metastasis.	It can mimic natural ECM. Inhibits tumor cell proliferations due to gel-forming properties at body temperature. Highly hydrophilic. Biocompatible and biodegradable.	Poor mechanical strength. Difficult to use in cell-based anticancer therapy due to poor cell adhesion properties.	[54]
Cellulose 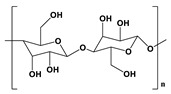	The glucose units are linked by glycosidic bonds and thereby form a polysaccharide structure.	Excellent mechanical properties. Hydrophilic in nature. Non-toxic.	Non-degradable.	[55]
Hyaluronic acid (HA) 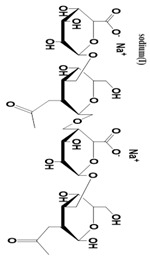	It consists of repeating disaccharide units. Presence of -OH and -COOH groups on the surface that can be chemically crosslinked with anticancer drugs.	High drug-loading properties. Facilitates tumor cell targeting properties. High degradable profile. Non-immunogenic.	Poor degradation profile. Unstable structure due to poor mechanical properties.	[56]
Glycosaminoglycans 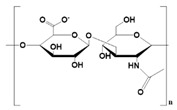	Individual disaccharide units are linked together by glycosidic bonds.	Anticancer activity. Prevents blood clots. Inhibits inflammatory pathway. Inhibition of metastasis.	Microbial Contamination.	[57]

**Table 2:** Synthetic materials used for preparing 3D scaffolds.

Synthetic Materials	SPR	Merits	Demerits	References
Polycaprolactone 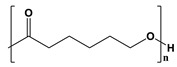	Presence of aliphatic ester chains.	It can block angiogenesis. High tensile strength. Plasticity. Biocompatible. Highly stable.	Low degradation profile. Hydrophobic.	[61]
Polylactic acid 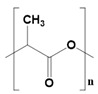	It contains -COOH as a functional group.	Excellent elastic properties. High mechanical properties. Thermally stable. Non-toxic. Hemocompatibility.	Hydrophobic. Non-degradable.	[62,63]
Polylactic-co-glycolic acid 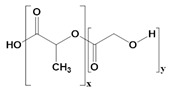	It is a block polymer of polylactic acid and polyglycolic acid.	High cellular interaction and migration. High mechanical properties. Tissue regeneration properties. Wound-healing properties. Enhance anticancer activities with doxorubicin.	Fragile structure. Poor tensile strength.	[64]
Polyglycolic acid 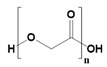	Linear polyester.	Hydrophilic. Can form nanoparticles. Thermal stability. Excellent tensile modulus.	Hydrolysis-based degradation.	[65]
Polypropylene fumarate 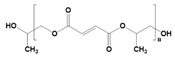	It consists of fumaric acid.	High mechanical strength. It can arrest the cell cycle in an abnormally grown cell lines. Biostable.	Viscous liquid.	[66]
Polyethylene glycol 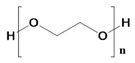	Derived from ethylene oxide	Highly elastic. Hydrophilic. Non-inflammatory. Mucoadhesive. Highly porous. Excellent polymers for targeted drug delivery system.	Poor cell interaction properties.	[67]
Polyurethane 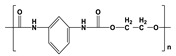	It consists of urethane groups.	High mechanical properties. Non-allergic. Thermally conductive. Heat resistance.	High hemolytic ratio. Less stable in biological environment.	[68]
Polyvinyl alcohol 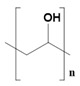	Polyhydroxy backbone.	It can mimic articular cartilages. Non-immunogenic. Hydrophilic. Hemocompatible.	Poor cell adhesion properties	[69]
Polypropylene Carbonate 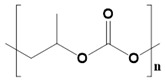	Block polymer of carbon dioxide and CH_3_CHCH_2_O.	High biodegradability. No inflammation. Structural stability. Non-toxic.	Rigid and fragile structure. Poor cell attachment.	[70]
Polyhydroxy butyrate 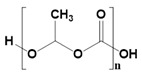	Beta-hydroxy acid. High crystallin structure.	Controlled release properties. Time-dependent degradation. Excellent candidate for drug delivery systems.	Highly rigid. Heat-induced instability.	[66]

**Table 3:** Next-generation 3D Scaffolds and their therapeutics outcome.

Types of Scaffolds and Polymers	Drugs	Route of Administration	Cell Line	Types of Cancer	Outcomes	References
LMW Chitosan and β-glycerophosphate	Doxorubicin	Intratumoral	H22 and SMMC 7721	Hepatoma	Consistent chemotherapy drug delivery to tumor tissue. Less toxicity to normal tissues.	[71]
Hyaluronic acid, Pluronic L121, and F127	Doxorubicin and Docetaxel	Intratumoral and peritumoral	CT-26	Colorectal carcinoma	Tumor inhibition. Reduce chemoresistance.	[72]
Polylactic-co-glycolic acid and polyethylene glycol	PLK1shRNA and Doxorubicin	Injection: beside tumors	Saos-2 and MG63	Osteosarcoma	Complete inhibition of cancer within 2 weeks. Higher apoptosis compared to single therapy. No systemic toxicity.	[73]
Poly(lactide-co-glycolide) and chitosan	Paclitaxel	Intratumor	M234-p	Mammary cancer	Crystal of paclitaxel decreases its action. A single dose of this scaffold is equal to four IP injections of paclitaxel. 63% of tumors suppressed. Non-toxic delivery system.	[74]
Polycaprolactone and polyethylene glycol	Porphyrin	Intravenous	HepG-2	Hepatocellular carcinoma	Excellent tumor targeting capability. Noninvasive. Biocompatible.	[75]
Polycaprolactone, 1,4,8-trioxa-spiro-9-undecanone, and polyethylene glycol	Doxorubicin, thermos-responsive NPs, and zinc phthalocyanine	Peritumoral	5637 cells	Bladder tumor	Less than 20% tumor cell viability after treatment. Less toxicity. Inhibits tumor growth.	[11]
Poly(ε-caprolactone) and polyethylene glycol	Paclitaxel	Subcutaneous	4T1	Breast cancer	Preventing primary breast cancer. Inhibits distal metastasis. Wound-healing properties.	[18]
Pluronic F127 and PECT	Nanocrystal of paclitaxel	Peritumoral injection	MCF-7	Breast tumor	High drug-loading efficiency. Long-time stable at peritumoral site. Comparable anticancer effects.	[76]
Chitosan, poly (N-isopropyl acrylamide-co-itaconic acid), and glycerophosphate	Doxorubicin	N/A	MCF-7	Breast cancer	Sustained drug release. Anti-proliferative effect.	[77]
Chitosan, dihydrocaffeic acid, and pullulan	Doxorubicin and amoxicillin	N/A	HCT116	Colon cancer and bacterial infections	Inhibits the proliferation of tumor cells. Antimicrobial properties. Good candidate for mucosal drug delivery.	[78]
LMW chitosan, cyclodextrin, and F127	Doxorubicin.	Intravenous	H22	Breast tumor	Complete regression of tumor. Target delivery to H22 tumor. No doxorubicin accumulation in healthy tissues.	[79]
Carboxyethyl chitosan and di-benzaldehyde polyethylene glycol	Doxorubicin	N/A	HepG2 and I929	Hepatocellular carcinoma	Self-healing properties. High drug-loading capacity. Long stability. Good cytocompatibility.	[80]
Polyethylene glycol methyl ether methacrylate and acrylic acid	5-Fluorouracil	N/A	HepG2 and LO2	Liver cancer	Controlled delivery of 5-Fluorouracil. Thermal, pH, and salinity sensitives.	[81]
Glycol chitosan, hyaluronic acid, and β-sodium glycerophosphate.	Doxorubicin	N/A	Hela	Cervical carcinoma	Excellent cancer cell adhesion. pH-sensitive drug release.	[82]
Polyacrylamide and DNA complex	Complementary DNA and doxorubicin	N/A	CEM	Lymphocytic leukemia	Maximum therapeutic response.	[83]
Poly-PPM	Platinum (IV) complex-mediated prodrug	Intravenous	A549	Lung cancer	Sustained drug release properties. Prolongs half-life. Oxygen-independent reactive oxygen species generation. High accumulation of drug in cancer cells. Downregulates the expression of multidrug resistance protein 1.	[84]

**Table 4:** Examples of the current scaffolds/hydrogels in clinical trials for cancer treatment (modified and reproduced under the terms of the Creative Commons CC BY license) [179].

Name (Sponsor Company/University)	Hydrogel Material/Payload (Gelation Mechanism)	Injection/Implant	Indications	Accessed on 1 October 2022 (http://clinicaltrials.gov) Identifier (Phase)
Absorbable Radiopaque Tissue Marker (Sidney Kimmel Comprehensive Cancer Center at Johns Hopkins)	Polyethylene glycol/TraceIT^®^ (chemical reaction)	Between pancreas and duodenum	Imaging of pancreatic adenocarcinoma	NCT03307564
Memorial Sloan Kettering Cancer Center	Polyethylene glycol (chemical reaction)	Visceral pleura	Lung biopsy	NCT02224924 (Ph III)
Absorbable Radiopaque Tissue Marker (Washington University School of Medicine)	Polyethylene glycol/TraceIT^®^ (chemical reaction)	Resection bed	Imaging of oropharyngeal cancer	NCT03713021 (Ph I)
Absorbable Radiopaque Hydrogel Spacer (Thomas Zilli, University Hospital, Geneva)	Polyethylene glycol/TraceIT^®^ (chemical reaction)	Between the target (prostate/vagina) and the organ (rectum)	Spacing in radiation therapy for rectal cancer	NCT03258541 (NA)
Augmenix, Inc.	Polyethylene glycol/SpaceOAR^®^ (chemical reaction)	Between the rectum and prostate	Spacing in radiation therapy for prostate cancer	NCT01538628 (Ph III)
Royal North Shore Hospital	Polyethylene glycol/SpaceOAR^®^ (chemical reaction)	Between the rectum and prostate	Spacing in radiation therapy for prostate cancer	NCT02212548 (NA)
University of Washington	Polyethylene glycol/TraceIT^®^ (chemical reaction)	Around circumference of the tumor bed	Imaging of bladder carcinoma	NCT03125226
Icahn School of Medicine at Mount Sinai	Polyethylene glycol/SpaceOAR^®^	Between the rectum and prostate	Spacing in radiation therapy for prostate cancer	NCT05224869 (Ph II)
Cancer applications: natural				
Gut Guarding Gel (National Cheng-Kung University Hospital)	Sodium alginate/calcium lactate (physical interaction)	Submucosal	Gastroenterological tumor and polyps	NCT03321396 (NA)
Smart Matrix Limited (Welsh Centre for Burns and Plastic Surgery, Swansea, UK Queen Victoria Hospital NHS Foundation Trust)	Human fibrin/alginate porous matrix	Surgical wound site	Basal Cell Carcinoma Squamous Cell Carcinoma	NCT02059252 (Ph I) (Ph II)
Smart Matrix Limited	Human fibrin/alginate porous matrix	Full-thickness wounds arising from surgical excision of basal cell or squamous cell carcinomas	Basal Cell Carcinoma Squamous Cell Carcinoma	NCT03742726 (NA)
Fibralign Corporation (University of Chicago Stanford University)	BioBridge^®^ Collagen Matrix	Upper limb lymphedema secondary to breast cancer treatment	Breast Cancer-Associated Lymphedema	NCT04606030 (NA)
Memorial Sloan Kettering Cancer Center (Integra LifeSciences Corporation)	(MatriStem PSM) A porcine-derived, extracellular matrix	Esophagus	Esophageal Adenocarcinoma	NCT01970306 (Ph II)

**Table 5:** Nanotechnology-based treatment approaches for cancer in clinical trials.

Nano-Chemotherapeutics	Type of Cancer	ClinicalTrials.gov Identifier
Carbon nanoparticles	Thyroid Cancer	NCT02724176
Docetaxel-PNP	Advanced Solid Malignancies	NCT01103791
Magnetic Nanoparticle Injection	Prostate Cancer	NCT02033447
TKM-080301	Colorectal Cancer with Hepatic Metastases	NCT01437007
	Pancreas Cancer with Hepatic Metastases	
	Gastric Cancer with Hepatic Metastases	
ExoIntelliScore Prostate	Prostate Cancer	NCT02702856
Dex2	Non-small Cell Lung Cancer	NCT01159288
ExoDx Prostate (IntelliScore)	Urologic Cancer	NCT04720599
IGF-1R/AS ODN	Malignant Glioma of Brain	NCT01550523
Etuximab nanoparticles	Colon Cancer, Colo-rectal Cancer	NCT03774680
Quercetin-encapsulated PLGA-PEG nanoparticles	Oral Cancer	NCT05456022
BIND-014	KRAS Positive Patients With Non-small Cell Lung Cancer	NCT02283320
SN-38 liposome	Colorectal Cancer	NCT00311610
Liposome Entrapped Docetaxel (LE-DT	Pancreatic Cancer	NCT01186731
Pegylated Liposomal Doxorubicin	Ovarian Neoplasms	NCT02751918
Rastuzumab and non-pegylated liposomal doxorubicin	Breast Cancer	NCT02562378
